# Neurosurgical education during COVID-19: challenges and lessons learned in Egypt

**DOI:** 10.1186/s41983-020-00242-8

**Published:** 2020-11-26

**Authors:** Ahmed Hamdy Ashry, Hussein Mohammed Soffar, Mohamed Fathalla Alsawy

**Affiliations:** Neurosurgery Department, Kasr Alainy Faculty of Medicine, Cairo University, Giza, Egypt

**Keywords:** COVID-19, Neurosurgical, Education, Developing, Countries

## Abstract

**Background:**

The coronavirus pandemic (COVID-19) has disrupted the routine neurosurgical education and practice worldwide and so more in developing countries. Continuing the neurosurgical training while maintaining the well-being of our residents should be the primary concern of leaders of training programs.

**Objectives:**

The aim of this cross-sectional study was the evaluation of the impact of COVID-19 on neurosurgical residency programs and neurosurgical practice in five tertiary medical centers in our country. We also aimed at detecting the shortcomings in training programs and provide solutions.

**Methods:**

An online questionnaire-based survey was prepared and sent to 73 neurosurgery residents in 5 tertiary centers in 4 governorates by social networks. The questions focused on the evaluation of clinical and surgical activities before and after the pandemic. Safety precautions, education, and residents’ mental health were also evaluated.

**Results:**

Fifty residents responded to our survey. We identified a significant reduction in surgical cases, inpatient services, and working hours per week during the pandemic comparing to the pre-pandemic era. We also identified a significant increase in research hours and changes in educational methods from in-person methods to virtual ones. Seventy-four percent reported that personal protective equipment was not adequate for their duties. Sixty-eight percent experienced burnout symptoms. Unavailability of personal protective equipment, negative concerns regarding the surgical career, and financial strains significantly affected the mental health of residents.

**Conclusions:**

The survey highlighted the negative impact of COVID-19 on neurosurgical practice and education. Being in a developing country, this negative effect was amplified due to financial reasons and weak infrastructure. Inadequate personal protective equipment increased the risk of infection and work-related stress among neurosurgery residents. We lacked telemedicine services in our country. Online education gained more visibility and awareness.

## Introduction

The COVID-19 pandemic left us handicapped regarding the circulation of both knowledge and skills. Moreover this pandemic shifted skilled medical personnel away from their areas of expertise [1]. Many neurosurgical training programs stopped their activities till subsidence of the crisis. Many solutions started to appear to compensate for learning difficulties [2]. In third world countries, matters became more complicated due to fragile healthcare systems, multiple deficiencies, and lack of funds [3].

In this study, we tried to assess the damage that has occurred to neurosurgery training programs in five tertiary medical centers. We also focused on evaluation of the residents’ mental health and the availability of safety precautions in light of deficiency of resources and wealth. We suggested some solutions which would help leaders of training programs to catch up with the delay that happened.

## Methods

We conducted this cross-sectional study via online voluntary 27-question survey which was sent to 73 neurosurgery residents in large five university hospitals in four governorates from June 1 to June 15, 2020. The survey was conducted using Google Forms (Google LLC, Mountain View, CA, USA) software. We contacted a senior resident in each university and sent him the link of the survey. He was asked to propagate it in various social media groups of neurosurgery residents in his institute. All questions were close-ended multiple choice and all the answers were anonymized to avoid bias (Table 1).

**Table 1:** The questionnaire

Questions	Answers
1. What is your residency year?	PGY1 PGY2 PGY3 PGY4 PGY5
2. How many surgical operations did you perform or assist in per week before the pandemic?	Less than 6 6–10 More than 10
3. How many surgical operations did you perform or assist in per week during the pandemic?	Less than 6 6–10 More than 10
4. Regarding your actual intraoperative role in the surgical steps, was it affected?	Yes, it was increased Yes, it was decreased No, unchanged
5. Did the number of residents scrubbed in the same surgical procedure decrease?	Yes No
6. Do you think that the pandemic would have a negative effect on your surgical skills?	Yes No Maybe
7. How many patients did you attend in the outpatient clinic per week before the pandemic?	Less than 30 30–49 50–79 80–100 More than 100
8. How many patients did you attend in the outpatient clinic per week during the pandemic?	Less than 30 30–49 50–79 80–100 More than 100
9. How many hours did you work per week before the pandemic?	Less than 50 50–59 hours 60–69 hours 70–80 hours More than 80
10. How many hours did you work per week during the pandemic?	Less than 50 50–59 hours 60–69 hours 70–80 hours More than 80
11. Were you deployed to other departments involved in COVID-19 patients’ management?	Yes No
12. How could you assess the role of neurosurgical residents in management of COVID-19 patients?	Of little clinical value Useful Critical
13. Did your institute provide telemedicine services for patients’ caring?	Yes No
14. Is ICU rotation a part of your training program at your institute?	Yes No
15. Do you think that the residency program needs to be extended?	Yes No
16. How many hours did you spend in research and academic work per week before the pandemic?	Less than 4 4–6 hours More than 6 hours
17. How many hours did you spend in research and academic work per week during the pandemic?	Less than 4 4–6 hours More than 6 hours
18. What was your main education method before the pandemic?	In person meetings and lectures Clinical staff rounds Webinars
19. What was your main education method during the pandemic?	In person meetings and lectures Clinical staff rounds Webinars
20. Did you test positive for COVID-19?	Yes No Not tested
21. Did you receive any instructions regarding the proper use of PPE?	Yes No
22. Was the PPE adequate for your duty?	Yes No
23. Did your institute provide a protocol for testing residents and other employees for COVID-19?	Yes No
24. Did you feel symptoms of burnout syndrome?	Yes No
25. Did you feel less or more support from your leaders and senior staff during the pandemic?	More support Less support Unchanged
26. Did you suffer from financial strains during the pandemic?	Yes No
27. Did the pandemic negatively affect your social life and personal relations?	Yes No

*PGY* postgraduate year, *PPE* personal protective equipment, *COVID-19* coronavirus disease 2019

Data were coded and entered using the Statistical Package for the Social Sciences (SPSS) version 26 (IBM Corp., Armonk, NY, USA). Junior residents were defined as those in postgraduate years 1–3 and senior residents as those in postgraduate years 4 and 5. For comparing categorical data, chi-squared (*χ*^2^) test was performed. Exact test was used instead when the expected frequency is less than 5. We categorized the data in two groups (pre-pandemic and pandemic). Comparisons between values measured before and after the pandemic were done using McNemar test. *P* values less than 0.05 were considered as statistically significant.

## Results

**Fig. 1:**
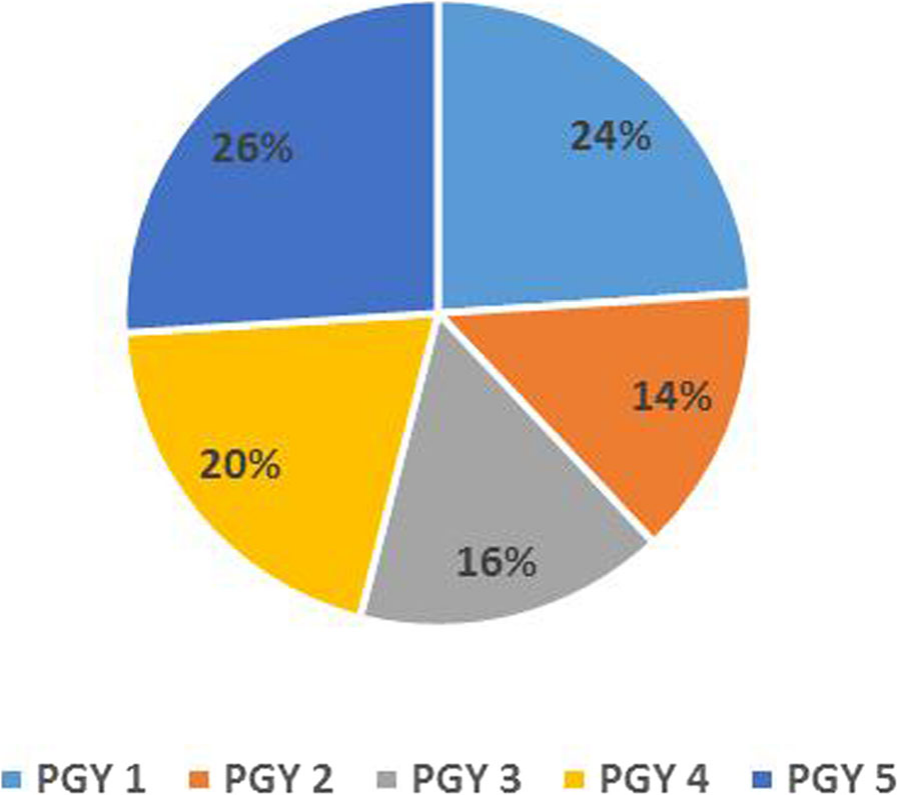
Postgraduate years (PGY) of the residents

We received responses from 50 residents in five neurosurgical tertiary centers in 4 governorates. Residents from all postgraduate years (PGY) were homogenously presented (Fig. 1). The survey included 27 junior residents (54%) and 23 senior residents (46%).

We identified a significant change in the volume and patterns of clinical and educational activities during the pandemic comparing to the pre-pandemic era (Table 2).

**Table 2:** Comparison between the pre-pandemic and pandemic era

Item		Before the pandemic, *n* (%)	During the pandemic, *n* (%)	*P* value
Surgical operations per week	< 6	5 (10)	37 (74)	< 0.001
	≥ 6	45 (90)	13 (26)	
Patients in OPC per week	< 30	3 (6)	38 (76)	< 0.001
	≥ 30	47 (94)	12 (24)	
Working hours per week	< 60	2 (4)	35 (70)	< 0.001
	≥ 60	48 (96)	15 (30)	
Research hours per week	< 4	41 (82)	8 (16)	< 0.001
	≥ 4	9 (18)	42 (84)	
Educational methods	In-person	35 (70)	6 (12)	< 0.001
	Virtual	15 (30)	44 (88)	

Twenty-eight residents (56%) reported reduction of their actual role in the surgical steps and reduction of the number of residents scrubbed in the same surgical procedure. Five residents (10%) were redeployed to other departments involved in the management of COVID-19 patients and all of them found their role of little clinical value. Twenty-four residents (48%) believed that the pandemic would have a negative effect on their surgical hand skills. The perception of this negative effect and the willingness to extend the training programs were more prevalent among senior residents (Table 3).

**Table 3:** Resident’ perception regarding the negative effect of the pandemic on the surgical skills and the extension of the training program across the residency year

Question		Residency year, *n* (%)		*P* value
		Senior (*n* = 23)	Junior (*n* = 27)	
Do you think that the pandemic would have a negative effect on your surgical skills?	Yes (*n* = 24)	19 (82.6)	5 (18.5)	< 0.001
	No (*n* = 18)	2 (8.7)	16 (59.3)	
	Maybe (*n* = 8)	2 (8.7)	6 (22.2)	
Do you think that the residency program needs to be extended?	Yes (*n* = 26)	21 (91.3)	5 (18.5)	< 0.001
	No (*n* = 24)	2 (8.7)	22 (81.5)	

*N* number of residents

Forty-five residents (90%) emphasized that their institutes were not ready to provide telemedicine services amid the crisis. All the residents reported that ICU rotation was not a part of their training programs.

Thirty-seven residents (74%) reported that personal protective equipment (PPE) was not adequate for their duties. Thirty residents (60%) did not receive any theoretical preparation regarding the proper use of PPE. In spite of defects in safety measures, only 4 residents (8%) were tested positive for COVID-19. Sixteen residents (32%) were tested negative and 30 residents (60%) were not tested. Forty residents (80%) reported that their institutes approved a protocol for testing residents and other employees.

Thirty-four residents (68%) experienced burnout symptoms and 31 residents (62%) suffered from financial strains during the pandemic. More than half of the participants believed the pandemic negatively affected their social life and personal relations. Twenty-seven residents (54%) reported that the support from their leaders remained unchanged comparing to the pre-pandemic period. Unavailability of PPE, financial strains, and residents’ perception regarding the negative effect of the pandemic on their hand skills were associated with high prevalence of burnout symptoms among residents (Table 4).

**Table 4:** Risk factors for burnout syndrome

Question		Burnout Symptoms, *n* (%)		*P* value
		Yes (*n* = 34)	No (*n* = 16)	
Residency year	Senior (*n* = 23)	16 (69.6)	7 (30.4)	0.827
	Junior (*n* = 27)	18 (66.7)	9 (33.3)	
Was the PPE adequate for your duty?	Yes (*n* = 13)	1 (7.7)	12 (92.3)	< 0.001
	No (*n* = 37)	33 (89.2)	4 (10.8)	
Did you suffer from financial strains during the pandemic?	Yes (*n* = 31)	31 (100)	0 (0)	< 0.001
	No (*n* = 19)	3 (15.8)	16 (48.2)	
Do you think that the pandemic would have a negative effect on your surgical skills?	Yes (*n* = 24)	21 (87.7)	3 (12.5)	< 0.001
	No (*n* = 18)	13 (72.2)	5 (27.8)	
	Maybe (*n* = 8)	0 (0)	8 (100)	

*N* number of residents

## Discussion

Many advanced neurosurgical centers in the developed world have responded promptly to the pandemic depending on the strength of their infrastructure and the abundance of their resources [4]. The matter was significantly different in the third world countries [5]. In our study, we tried to highlight the impact of COVID-19 pandemic on the neurosurgery residents in five large neurosurgical centers in our country. We also tried to discover deficiencies in our training programs and to develop future solutions.

The obvious reduction in surgical cases was due to canceling elective surgeries and decreasing the flow of patients [6]. Patients in rural areas found difficulties in accessing healthcare services in urban areas due to the lockdown and curfew imposed by the authorities. The number of residents scrubbed in the same surgical procedure was decreased to the minimum to preserve PPE’s and limit the contact. Senior residents expressed more concern over this point because they usually played a more active role in the surgical work.

We recommend future plans to resume the surgical activity after sustained reduction in the number of new COVID-19 cases. We suggested extending the schedule horizontally by increasing the time of elective surgery beyond the official working hours. We also recommend providing funds for “Super Friday” strategy which is a special weekend for performing high surgical volume. This approach can increase the productivity and surgical exposure of residents in a tight timeframe and act as boosting surgical courses. The residents who missed certain rotations in less urgent subspecialties should be given compensatory rotations. In our survey, most senior residents approved a proposed plan to extend the training program before applying for the board exams. Junior residents were less worried as they believed they still had the time to make up for this shortage.

In our country, telemedicine faces a lot of challenges related to the weak infrastructure, poor funding, resistance to change, and high illiteracy [7]. It is a good method of continuing the clinical education without in-person contact with patients. We reported a primitive attempt during the early time of the pandemic to provide postoperative care to neurosurgical patients in one of our referral hospitals using smartphones and popular social media applications [8]. But this attempt was dependent on individual efforts, not on institutional work.

Hands-on skills are the heart of any surgical practice. Therefore, continuing the surgical training while maintaining the well-being of our residents should be the primary concern of leaders of training programs. We noticed significant awareness of virtual methods of education during the pandemic which helped compensate for the decrease in surgical volume. In developing countries, residents find many obstacles in traveling abroad to attend neurosurgical conferences and hands-on workshops due to poor funding from their institutes [9]. Virtual methods of education provided a cost-effective method of interaction with international distinguished speakers.

In our country, neurosurgery residents are overwhelmed by secretarial and parasurgical work that could be conducted by paramedical staff. Also hospital administrations are concerned with delivery of neurosurgical service more than scientific research [10]. The majority of residents reported a significant increase in the number of hours spent in research thanks to the reduction in the work load in operating theaters during the pandemic.

Exams are important part of the educational process. Postponing the board exams was inevitable in these extraordinary circumstances. Implementation of computer-based platforms to provide online exams may take time. So we recommend holding face to face exams under strict precautions such as screening the candidates for fever at the entrance, reducing the exam time and social distancing in the exam centers.

In our survey, 10% of neurosurgery residents reported their redeployment to help treat the rising numbers of COVID-19 patients and they emphasized that their redeployment to the frontlines was of little clinical value.

Unlike USA and Europe, ICU rotation is not a part of neurosurgical training in our programs [5]. COVID-19 hit our country few months later after Wuhan. Unfortunately, we did not use this time to provide extra training to our residents to work outside their areas of expertise. Redeployment plans were prepared in haste without adequate supervision or much regard for each doctor’s knowledge or mental health. Many residents were not paid for the additional working hours. Many residents also complained of inadequate testing which raised their fears about being infected and transmitting infection to their families. Redeployment in most hospitals was obligatory to make up for the shortage of doctors. The public health system in our country faces the problem of shortage of physicians because of their emigration searching for better working conditions [11]. This shortage was exacerbated during the pandemic due to the increased rate of infection among the medical staff and the tsunami of COVID-19 patients especially in the early time of the pandemic when the health authorities decided to isolate all COVID-19-positive cases, even mild or asymptomatic, in the hospitals.

As documented in other surveys, 68% of residents experienced burnout symptoms with no significant difference between seniors and juniors [12]. In developing countries, residents are susceptible to more stress as they are forced to work under suboptimal conditions with less financial reward [13]. In the time of COVID-19 pandemic, this stress was amplified due to financial strains, shortage of PPE, and residents’ negative expectations regarding their surgical career. They were forced to buy their own PPE. In our country, many residents worked in private hospitals alongside university hospitals to increase their income [11]. Many private hospitals were designated for isolation and management of coronavirus patients, and the rest of other disciplines became marginalized. Many neurosurgery residents lost their jobs in these hospitals, which was a fundamental source of income.

In our institutes, many faculty members exploited their relationships with trustworthy charities to provide donations to buy PPE. Using the spirit of innovation, some faculty members were able to design a face shield and distribute it to the residents for free. “Talk to your boss” is a key action in this critical climate. Emotional support and engagement of the residents in setting future plans may reassure them about their careers.

## Conclusions

The COVID-19 pandemic has disrupted the routine neurosurgical practice in our country. Providing additional surgical courses and postponing board exams are suggested solutions to compensate for the reduction in clinical activities. Providing adequate PPE and financial and psychological support is very critical to the physical and mental health of residents. Implementation of telemedicine projects is no longer a luxury and helps preserve time and resources. Increasing the awareness of virtual methods of medical education is a bright aspect of the pandemic.

## Data Availability

The dataset used and analyzed during the current study are available from the corresponding author on reasonable request.

## Abbreviations

COVID-19: Coronavirus disease 2019
PPE: Personal protective equipment
PGY: Postgraduate year
OPC: Outpatient clinic
SPSS: Statistical Package for the Social Sciences
USA: United States of America
ICU: Intensive care unit
